# Detection of Species-Specific Lipids by Routine MALDI TOF Mass Spectrometry to Unlock the Challenges of Microbial Identification and Antimicrobial Susceptibility Testing

**DOI:** 10.3389/fcimb.2020.621452

**Published:** 2021-02-04

**Authors:** Vera Solntceva, Markus Kostrzewa, Gerald Larrouy-Maumus

**Affiliations:** ^1^ MRC Centre for Molecular Bacteriology and Infection, Department of Life Sciences, Faculty of Natural Sciences, Imperial College London, London, United Kingdom; ^2^ Bruker Daltonik GmbH, Bremen, Germany

**Keywords:** matrix-assisted laser desorption ionization, lipids, diagnostics, drug resistance, gram-negative, gram-positive, mycobacteria

## Abstract

MALDI-TOF mass spectrometry has revolutionized clinical microbiology diagnostics by delivering accurate, fast, and reliable identification of microorganisms. It is conventionally based on the detection of intracellular molecules, mainly ribosomal proteins, for identification at the species-level and/or genus-level. Nevertheless, for some microorganisms (e.g., for mycobacteria) extensive protocols are necessary in order to extract intracellular proteins, and in some cases a protein-based approach cannot provide sufficient evidence to accurately identify the microorganisms within the same genus (e.g., *Shigella* sp. vs *E. coli* and the species of the *M. tuberculosis* complex). Consequently lipids, along with proteins are also molecules of interest. Lipids are ubiquitous, but their structural diversity delivers complementary information to the conventional protein-based clinical microbiology matrix-assisted laser desorption ionization time-of-flight (MALDI-TOF) based approaches currently used. Lipid modifications, such as the ones found on lipid A related to polymyxin resistance in Gram-negative pathogens (e.g., phosphoethanolamine and aminoarabinose), not only play a role in the detection of microorganisms by routine MALDI-TOF mass spectrometry but can also be used as a read-out of drug susceptibility. In this review, we will demonstrate that in combination with proteins, lipids are a game-changer in both the rapid detection of pathogens and the determination of their drug susceptibility using routine MALDI-TOF mass spectrometry systems.

## Introduction

In recent years, matrix-assisted laser desorption ionization/time-of-flight (MALDI-TOF) mass spectrometry (MS) has revolutionized the field of microbiology (Clark et al., 2013; Singhal et al., 2015). MALDI-TOF MS provides rapid, accurate and cost-effective identification of a wide range of microbes based on protein signatures and requires only a single small colony for analysis (Croxatto et al., 2012). Development of commercial MALDI-TOF MS platforms capable of microbial identification and their subsequent approval for clinical use has made MALDI-TOF MS the standard routine identification tool in most diagnostic laboratories. Despite the advantages of MALDI-TOF MS, there are still several limitations to identification by protein profiling, such as the inability to differentiate closely related species and the laborious sample preparation required for some microorganisms.

In addition to proteins, lipids are also major cellular constituents. Lipids display high structural diversity and complexity, including species-specific characteristics which enable them to act as useful biomarkers for microbial identification. Although lipids have been used to characterize microorganisms since the 1960s (Abel et al., 1963; Moss and Lewis, 1967; Moss et al., 1980), the initial gas chromatography methods used for lipid analysis were time-consuming and unsuitable for clinical use. In the last decade, the popularity of microbial lipidomics has soared as novel methods of lipid analysis using MALDI-TOF MS have been developed (Cox et al., 2015; Larrouy-Maumus and Puzo, 2015; Leung et al., 2017). These methods can provide rapid and accurate microbial identification and differentiation and have the potential to address some of the challenges encountered by the proteomic approach and thereby complement them. Furthermore, analysis of lipids by MALDI-TOF MS is a promising tool for the detection of antibiotic resistance, particularly in the case of rapidly spreading resistance to polymyxins.

This review starts with a short overview of routine MALDI-TOF MS analysis and some of its current constraints, followed by the introduction of species-specific lipids and a brief history of lipid analysis for microbial identification. Afterwards, we will discuss the recent applications of mass-spectrometry based lipidomics for identification of microorganisms and detection of antibiotic resistance. We finish with an outlook toward the future directions for MALDI-TOF MS in microbial lipidomics.

## Protein-Profiling by MALDI-TOF for Microbial Identification

In recent years, analysis of intracellular proteins signatures by MALDI-TOF MS has become a routine tool for the characterization of microorganisms (Croxatto et al., 2012; Angeletti and Ciccozzi, 2019; Bryson et al., 2019). Two commercial MALDI-TOF MS platforms, the MALDI Biotyper (Bruker Daltonics Inc.) and the VITEK MS (bioMérieux Inc.) - both recently approved by the FDA - are used in the majority of microbiology laboratories (Patel, 2015). For many microorganisms, these platforms enable high-throughput identification using simple sample preparation procedures and requiring only a single colony. Some microbes can be identified using direct cell profiling in which a colony is smeared on a MALDI target plate followed by the addition of a MALDI matrix that extracts the intracellular, mainly ribosomal, proteins (van Veen et al., 2010). For other microorganisms, including Gram-positive bacteria, a simple protocol of protein extraction using formic acid is sufficient prior to MALDI-TOF MS analysis (Bizzini et al., 2010; Alatoom et al., 2011).

Following sample processing, the protein mass spectra of an unknown isolate are acquired in the mass range of *m*/*z* 2,000 to *m*/*z* 20,000 in the positive ion mode. This range includes the ribosomal proteins that are present at high abundance in the cell. The mass spectra are then compared to a database that contains the profiles of known microbial species (Murray, 2012). As verified in numerous studies, MALDI-TOF MS provides highly accurate and reliable identification of a wide range of microorganisms (Cherkaoui et al., 2010; Carbonnelle et al., 2012; Wilson et al., 2017).

In addition to bacteria, MALDI-TOF MS is a valuable tool for the identification of fungal pathogens, particularly pathogenic yeasts (Buchan and Ledeboer, 2013). Many medically important yeast species, including *Candida* species and *Cryptococcus neoformans*, can be rapidly identified using simple on-plate extraction with formic acid (Theel et al., 2012; Westblade et al., 2013). MALDI-TOS MS identification of filamentous fungi can also be achieved but is not currently as successful as yeast typing due to the presence of robust cell walls and a lack of comprehensive fungal reference libraries (Becker et al., 2014; Wilkendorf et al., 2020).

Although protein profiling by MALDI-TOF MS is a valuable tool in microbiology, it has several constraints that will need to be addressed in the future. For microorganisms that are encased in complex, thick cell walls, laborious protein extraction procedures are required (Marklein et al., 2009; Rodriguez-Temporal et al., 2018; Kostrzewa et al., 2019). For example, a multistep method that involves heat inactivation and protein extraction is used for mycobacteria due to their robust cell walls and biosafety concerns (Wilen et al., 2015). According to the specimen preparation protocol described by El Khéchine et al., mycobacteria are first heat-inactivated for 1-hour at 95°C. Afterwards, mycobacterial cell walls are disrupted by vortex mixing with glass beads and the proteins are extracted using formic acid and acetonitrile (El Khechine et al., 2011). Labor-intensive protocols involving protein precipitation with ethanol and subsequent extraction with formic acid and acetonitrile are also used for yeasts to improve identification results (Bader, 2017).

Another major constraint of MALDI-TOF MS is a commonly encountered failure to differentiate some closely related species (Bizzini et al., 2010; Alcaide et al., 2018). For example, MALDI-TOF MS can fail to differentiate *Shigella* from *E. coli* (Martiny et al., 2012). These Gram-negative bacteria are very closely related and could genotypically be considered as the same species, thus explaining the problems of differentiation using protein fingerprinting. Rapid discrimination between the two species is extremely important for infection control because *Shigella* species can cause shigellosis, an acute intestinal infection that is highly contagious. Enteroinvasive *E. coli* may trigger similar symptoms, but the disease is significantly less communicable (Niyogi, 2005). It also remains challenging to differentiate other closely related microorganisms such as the *Mycobacterium tuberculosis* complex which includes *M. africanum*, and the animal-adapted *M. caprae*, *M. bovis*, *M. microti*, *M. pinnipedii*, and *M. canettii*, or the *M. abscessus* complex which is composed of *M. abscessus* subsp. *abscessus*, *M. abscessus* subsp. *bolletii*, and *M. abscessus* subsp. *massiliense* (Saleeb et al., 2011; Nessar et al., 2012; Benwill and Wallace, 2014; Johnson and Odell, 2014; Angeletti et al., 2015; Bar-On et al., 2015). Other examples of erroneous identification of closely related species include *Streptococcus mitis* and *Streptococcus pneumoniae*, *Bordetella pertussis* and *Bordetella bronchiseptica*, *Klebsiella pneumoniae* and closely related species/subspecies, *Haemophilus influenzae* and *Haemophilus haemolyticus* and members of the *Enterobacter cloacae* complex (*Enterobacter cloacae*, *Enterobacter asburiae*, *Enterobacter hormaechei*, *Enterobacter kobei*, *Enterobacter ludwigii* and *Enterobacter nimipressuralis*, *Enterobacter. cloacae* and *Enterobacter hormaechei* (Stevenson et al., 2010; Frickmann et al., 2013; Angeletti et al., 2015; Schulthess et al., 2016; Rodrigues et al., 2017).

Consequently there is an ongoing need for the manufacturers of MALDI-TOF MS systems to continually update the databases and libraries with new reference spectra as new pathogens emerge innovative sample preparation improves microorganism identification. In addition to these commercial databases research laboratories can create custom reference spectra and expand existing databases with new microorganisms and applications. However, benchmarking against a reference method such as whole genome or 16S rRNA sequencing is required to ensure accurate identification and avoid misassignments.

## Lipids as Species-Specific Biomarkers

Lipids are also a major functional and structural constituent of cells. and play important roles in membrane formation, cell signalling, energy storage and cell recognition (Hannun and Obeid, 2008; van Meer et al., 2008; Nakamura et al., 2014). To perform such diverse functions, cells contain a variety of lipid molecules that differ in the nature of the backbone and the headgroups, the number of fatty acids, and the chemical moieties they are modified with. Fatty acids themselves also vary in the length of the chain and the number of double bonds (Fahy et al., 2005). In combination with proteins, the diversity of lipids has the potential to make them useful biomarkers for microbial identification.

The differences in the cell wall structure and the membrane composition between Gram-positive and Gram-negative bacteria are well established, however, the lipid composition also varies among the species belonging to the same Gram type (Sohlenkamp and Geiger, 2016). In addition to common glycerophospholipids such as phosphatidylethanolamine and phosphatidylglycerol that are found in many organisms, bacteria possess species-specific lipids that display a high level of structural variability and are particularly useful for species differentiation. In Gram-negative bacteria, lipopolysaccharide (LPS) is a major structural component of the outer membrane. It is a glycolipid comprised of three parts: lipid A, a hydrophobic section of the molecule responsible for some aspects of toxicity of Gram-negative bacteria, the hydrophilic core oligosaccharide and the O antigen, a polysaccharide comprising the outermost domain of LPS (Raetz and Whitfield, 2002). The structure of LPS varies significantly among different species with the O antigen being the most diverse part of the molecule (Lerouge and Vanderleyden, 2002). The structure and composition of the O antigen can vary between strains of one species and has been used to assign serotypes of *Salmonella* and *E. coli* (Orskov and Orskov, 1992; Liu et al., 2014). Casabuono et al., reported on the structural characterization of lipid A from *Shigella flexneri* variant X, and discrete variants could serve to discriminate *Shigella* species from *E. coli* (Casabuono et al., 2012).

Gram-positive bacteria contain glycolipids, glucolipids and lipoteichoic acid (LTA), which is a typical constituent of the cell membrane (Reichmann and Grundling, 2011). LTA is defined as an alditol phosphate-containing polymer that is linked by a lipid anchor to the membrane. Based on chemical structure, there are five types of LTA (types I–V) (Percy and Grundling, 2014). Type I LTA is the most frequently encountered polymer found in many Gram-positive bacteria belonging to the phylum Firmicutes, including *B. subtilis*, *S. aureus* and *S. pyogenes* (Schneewind and Missiakas, 2014). Even though all type I LTA molecules have a common polyglycerol-phosphate backbone, variations of the core structure have been described in different species, particularly in the glycolipid anchor attaching the LTA to the membrane (Roethlisberger et al., 2000; Shiraishi et al., 2013). Type II and III LTA molecules, found in *Lactococcus garvieae* and *Clostridium innocuum*, respectively, contain repeat units of glycosylalditol-phosphate (Koch and Fischer, 1978). Type IV LTA is a complex molecule found in *S. pneumoniae* and some other *Streptococcus* species (Fischer, 1997).

In mycobacteria, several species-specific lipids have been described (Alberghina, 1976; Brennan and Nikaido, 1995; Ortalo-Magne et al., 1996; Ripoll et al., 2007; Batt et al., 2020). Mycolic acids are unique long-chain fatty acids that comprise the inner leaflet of the external mycomembrane in mycobacteria (Jackson, 2014). Mycolic acids display high structural diversity with variations observed in the chain length (60 to 90 carbon atoms), the level of unsaturation, and the chemical groups, such as ketones and methoxys (Marrakchi et al., 2014). This high level of diversity provides mycolic acids with species-specific characteristics making it possible to use them as taxonomic markers (Song et al., 2009). In addition to mycolic acids, other lipids are also found to be specific to particular species of mycobacteria. For example, sulfolipid 1 and polyacyltrehalose are found exclusively in *M. tuberculosis*, while trehalose polyphleate is present in non-tuberculosis mycobacteria (NTM) species (Hatzios et al., 2009; Layre et al., 2011; Burbaud et al., 2016).

Glycolipids can also be used for the identification of fungi. Glycosylinositol-phosphorylceramides (GIPCs), which are a class of glycosphingolipids uniquely found in plant and fungal species, are particularly important biomarkers due to their high structural diversity (Fontaine et al., 2003; Costachel et al., 2005; Simenel et al., 2008; Saromi et al., 2020). GIPCs are major players in the establishment of infection in the vertebrate host by filamentous fungi such as *Aspergillus fumigatus* since they are involved in adhesion, signal transduction, modulation of the host immune response, and apoptosis (Toledo et al., 2007; Guimaraes et al., 2014; Fernandes et al., 2018). The conserved core structure of GIPCs consists of a ceramide moiety attached to a glucuronic acid-inositolphosphate group. Many diverse saccharides can be added to this core structure which generates complex glycan arrangements and results in significant structural variation among different fungal species (Cacas et al., 2012; Bure et al., 2014; Guimaraes et al., 2014).

As well as lipids from Gram-negative bacteria, Gram-positive bacteria, mycobacteria and filamentous fungi lipids, archaebacteria lipids have the potential to provide lipid-specific biomarkers. In contrast to glycerol-ester phospholipids from bacteria and filamentous fungi archaebacterial membranes are composed of isoprenoid and hydroisoprenoid hydrocarbons and isopranyl glycerol-ether lipids (De Rosa et al., 1986). Although these structures vary according to phenotypes (e.g., halophiles, methanogens, and thermophiles), these molecules are formed by condensation of glycerol or more complex polyols with isoprenoid alcohols containing 20, 25, or 40 carbon atoms (Woese et al., 1978; De Rosa et al., 1986). Indeed, due to their heterogeneity, these lipids have been proposed for the identification and classification of archaebacteria (Tornabene et al., 1980). They can therefore serve as chemical markers in combination with conventional 16S rRNA sequencing (McCartney et al., 2013; Sollai et al., 2019a; Sollai et al., 2019b).

As far as we are aware, routine identification of microorganisms by MALDI-TOF MS based on the identity and abundance of lipids has not been explored extensively to differentiate species and subspecies. This represents an exciting area which deserves more investigation.

## History of Microbial Lipid Analysis

Early attempts to utilize lipids for microbial identification were based on the analysis of fatty acids released from the microbial cells by chemical treatments and their subsequent analysis using either gas chromatography (GC) or gas chromatography mass spectrometry (GC/MS) (Abel et al., 1963; Moss and Lewis, 1967; Moss et al., 1980; Athalye et al., 1985; Kotilainen et al., 1991; Osterhout et al., 1991; Butler and Guthertz, 2001; Mosca et al., 2007). Both lipid and fatty acid composition varies among microbial species. Although most bacteria synthesize fatty acids that have chain lengths of 10 to 19 carbons, the relative abundance of each type of fatty acid varies among different species (O’Leary, 1962). Furthermore, some fatty acids are restricted to particular species such as the internally branched iso-fatty acids found in *Gaiella occulta* (Albuquerque et al., 2011). As a result of the quantitative differences in the fatty acid content and the presence of unusual or rare fatty acid types, the fatty acid profile of each species is distinctive which enables it to act a taxonomic marker.

The first evidence that bacteria can be identified based on their fatty acid composition was presented by Abel et al. in 1963. Their study employed gas chromatography to analyse the fatty acid composition of bacteria belonging to the family *Enterobacteriaceae* and some Gram-positive species (Abel et al., 1963). The major cellular fatty acids with chain lengths of 9 to 22 carbon atoms were extracted and trans-esterified into more volatile fatty acid methyl esters, followed by gas chromatographic analysis. The chromatographic profiles acquired were found to be distinct for each species which enabled bacterial characterization (Abel et al., 1963).

Subsequent studies have employed fatty acid analysis by gas chromatography for species-level identification of Gram-positive and Gram-negative bacterial species, mycobacteria and fungi (Jantzen et al., 1989; Veys et al., 1989; Marumo and Aoki, 1990; Kotilainen et al., 1991). Characterization was based on fatty acids with chain lengths between 9 and 20 carbon atoms, which represent the highly abundant fatty acids of phospholipids and glycolipids (Welch, 1991). Fatty acid analysis was particularly useful for identification of non-fermentative Gram-negative bacteria which are challenging to identify using conventional biochemical tests due to their relative un-reactivity (Veys et al., 1989; Wallace et al., 1990).

In 1991, efforts to automate and standardize the method were made which led to the introduction of the Sherlock Microbial Identification System (MIS). The MIS is an automated gas chromatographic system that identifies microorganisms based on the analysis of fatty acids that are between 9 and 20 carbons in length. The MIS automatically names and quantitates fatty acids enabling computerized identification of bacteria and fungi by comparing the fatty acid profile of the sample to profiles of known species in the database. It achieved a relatively high accuracy of identification for a range of microorganisms, including mycobacteria and non-fermentative Gram-negative bacteria (Osterhout et al., 1997; Mosca et al., 2007).

Despite the popularity of fatty acid analysis, this method has not reached clinical microbiology laboratories due to several limitations. The major drawback of gas chromatographic analysis of fatty acids is the time-consuming and laborious sample preparation. Before gas chromatography analysis, fatty acids are released by saponification and then methylated to generate fatty acid methyl ester (FAME) derivatives. FAMEs have increased volatility which facilitates their analysis by gas chromatography (Welch, 1991). This requires a multistep protocol which involves saponification of bacterial cells in sodium hydroxide, methylation with hydrochloric acid, lipid extraction and a wash at a basic pH. The procedure requires sufficient technical expertise and typically takes several hours (Mosca et al., 2007). Another constraint of the method is how fatty acid composition varies depending on cell growth conditions such as temperature, medium composition and growth phase (Marr and Ingraham, 1962). To obtain a reproducible fatty acid profile, growth conditions must be standardized with great care, otherwise changes in fatty acid composition may lead to incorrect identification when the profile is compared to the reference database.

Analytical techniques including electrospray ionization (ESI) MS and Direct Analysis in Real Time (DART) MS have also been used as alternatives to GC-MS for fatty acid profiling (Song et al., 2009; Cody et al., 2015). However, attention largely shifted away from lipid analysis and those analysis techniques when MALDI platforms entered clinical laboratories and the proteomic approach became the most practical and rapid method of microbial identification.

## Lipid-Profiling by MALDI-TOF for Microbial Identification

MALDI-TOF MS is a powerful analytical tool with applications in microbiology not limited to protein profiling. Over the last decade many groups have developed MALDI-TOF routines for monitoring the degradation of antibiotics such as carbapenems and indicators of antibiotic resistance (Hrabak et al., 2011; Dortet et al., 2018c; Kostrzewa, 2018; Anantharajah et al., 2019; Huang et al., 2020). Several research groups have also employed MALDI-TOF MS for the analysis of lipid profiles from microorganisms (Cox et al., 2015; Larrouy-Maumus and Puzo, 2015; Leung et al., 2017; Liang et al., 2019).

Cox et al. developed a novel method of fatty acid analysis that uses CeO2-catalyzed fragmentation of lipids to produce fatty acids using the energy inherent to the MALDI laser (Cox et al., 2015). The conversion occurs *in situ* as the lipid extracts are added directly to the MALDI target plate spotted with CeO2. The technique, termed metal oxide laser ionization (MOLI) MS, obtained reproducible species- and strain-specific fatty acid profiles of isolates belonging to *Enterobacteriaceae*, *Acinetobacter* and *Listeria* genera. The accuracy of the method, validated by multivariate statistical methods, reached 100% for species-level classification and 98% for strain-level classification with only one strain being identified incorrectly. Particularly important for clinical microbiology, MOLI MS correctly identified the isolates of *Shigella*, the bacteria routinely classified by protein profiling as *E. coli* (Cox et al., 2015). A subsequent study that analyzed 50 *Staphylococcus* isolates including *S. aureus* strains and other species demonstrated a high accuracy of species- and strain-level identification (Saichek et al., 2016). Despite these initial promising results, this technology has not yet arrived in routine microbiology laboratories due to the constrains related to *in vitro* diagnostics, accessibility and implementation to such technology in clinical laboratories world-wide

Although fatty acids were traditionally used for microbial identification, analysis of whole lipids can be a more informative approach. Lipids are more complex molecules with greater scope for structural diversity and are therefore more suited to act as a chemical fingerprint for differentiation of microorganisms. A novel method of bacterial identification based on the analysis of glycolipids was recently developed by Leung et al. (2017). Glycolipids are a particularly diverse class of lipids due to the large variety of sugar modifications which enables them to generate species-specific mass spectral profiles. Furthermore, bacterial glycolipids are exclusive to bacterial cells and are not produced by mammals, which facilitates direct identification of bacteria from biological fluids. By analyzing the lipid extracts using MALDI-TOF MS, Leung and co-workers demonstrated that glycolipid mass spectra enabled differentiation of ESKAPE pathogens, a clinically relevant group of bacteria that have increased resistance to antibiotics. In addition, they employed the existing MALDI Biotyper software to construct a glycolipid library containing 50 microbial entries (Leung et al., 2017). This dataset was used in the subsequent machine learning study to identify *A. baumannii* and *K. pneumoniae* from polymicrobial mixtures, such as urinary tract infection specimens (Fondrie et al., 2018).

Traditionally, prior to MALDI-TOF MS analysis, lipid extraction is necessary. Leung et al. used a microextraction protocol developed by El Hamidi et al. which extracts glycolipids using hot ammonium isobutyrate (El Hamidi et al., 2005). Despite being efficient, this method is time-consuming and uses hazardous chemicals which limits its clinical utility. More recently, Liang et al. (2019) developed a novel method of lipid extraction in which sodium acetate buffer is added to the bacterial sample, followed by brief heating and organic solvent extraction. Using this method, bacterial identification can be completed in less than an hour making it a promising method for clinical diagnostics (Liang et al., 2019).

As mentioned previously, analysis of lipids or fatty acids required extraction of these molecules from the cells such as organic solvents extraction, partition and concentration (Bligh and Dyer, 1959; Maddi, 2019). Once extracted, those lipids could be analyzed by MALDI-TOF MS with the appropriate matrix to enable their ionization and desorption. For example, Angelini et al. have used 9-aminoacridine (9-AA), a matrix originally developed for the rapid analysis of glycerophospholipids, for direct analysis of lipid from extremely halophilic archaeon *Halobacterium salinarum* (Sun et al., 2008; Angelini et al., 2010). Prior to MALDI-TOF MS analysis, the lyophilized purple membrane of *H. salinarum* was finely ground with the dry 9-AA matrix and crushed in a mechanical die press. The resulting pellet was applied to the MALDI target and analyzed. This work showed that the 9-AA matrix enabled good ionization and easy detection of archaeal phospholipids and cardiolipins. The lipid profile generated using this method corresponded well to the MALDI-TOF MS profile obtained from the lipid extract of *H. salinarum*, demonstrating that it is possible to perform direct lipid analysis of intact lyophilized archaebacterial membranes, without the need for lipid extraction (Angelini et al., 2010).

9-AA matrix has also been utilized for lipid analysis of intact viruses (Vitale et al., 2013). Purified viral suspensions were spotted on the MALDI target, followed by water evaporation and application of 9-AA matrix solution. The evaporated samples were then directly analyzed by MALDI-TOF MS. This method not only allowed the detection of the major lipids of these viruses, but also revealed novel details about the viral membrane composition, such as the presence of minor amount of phosphatidylcholine in PRD1 and φ6 bacteriophages (Vitale et al., 2013).

Another MALDI matrix, 1,8‐bis(dimethylamino)naphthalene (DMAN), has also been utilized for lipid fingerprinting of intact Gram-positive *Lactobacillus sanfranciscensis* and *Lactobacillus planta-rum* strains (Calvano et al., 2011). The use of DMAN, which is a highly basic matrix, improves the detection of low molecular weight, less ionizable phospholipids, such as glycolipids and cardiolipins, in the negative ion mode due to the complete absence of matrix-related signals which can otherwise preclude lipid detection (Calvano et al., 2011).

However, a novel method developed by Larrouy-Maumus et al. enables direct MALDI-TOF MS analysis of lipids on intact microbes (Larrouy-Maumus and Puzo, 2015). A major advantage of this approach is a simple, rapid sample preparation that does not require any chemical treatment or purification prior to MALDI-TOF MS analysis. Heat-inactivated microorganisms are washed three times in double-distilled water and deposited on the MALDI target plate, followed by the specific MALDI matrix. The matrix consists of a 9:1 mixture of dihydroxybenzoic acid and 2-hydroxy-5-methoxybenzoic acid (super-DHB) solubilized in apolar solvent system (Larrouy-Maumus and Puzo, 2015; Larrouy-Maumus et al., 2016). The super-DHB matrix was chosen due to its versatility for the analysis of phospholipids and lipids (Schiller et al., 2007). Using this approach, microbial identification can be completed in less than 10 minutes on fewer than 1,000 bacteria making it a useful tool in the clinical laboratory (Gonzalo et al., 2020). To date, this method has been used to differentiate mycobacteria, filamentous fungi and detect lipid A from Gram-negative bacteria (Dortet et al., 2018a; Dortet et al., 2018b; Furniss et al., 2019; Dortet et al., 2020a; Dortet et al., 2020b; Furniss et al., 2020; Saromi et al., 2020). In the case of mycobacteria, (excluding uninterpretable results where 38/273 (14%) of isolates could not be assigned to either the Mtb or the NTM group, and 9 isolates (3%) were misidentified) this method not only achieves accurate identification with a sensitivity and specificity of 96.7% and 91.7%, respectively, but also represents a bio-safe alternative to conventional MALDI-TOF MS with minimal sample handling and preparation (Gonzalo et al., 2020).

## Lipid MALDI for Bacterial Drug Susceptibility

As antibiotic-resistant bacteria emerge worldwide, the timely detection of antibiotic resistance is critical for appropriate therapeutic management and effective infection control. One of the most promising applications of lipid-based MALDI-TOF MS analysis is the detection of colistin resistance. Colistin is one of the polymyxin antibiotics used as a last-resort therapy for treating multidrug-resistant Gram-negative bacterial infections (Poirel et al., 2017). Resistance to colistin most commonly arises from chemical modifications of the lipid A portion of LPS and can be mediated through chromosomal mutations or by the activity of plasmid-encoded mobile colistin resistance (MCR) genes. These modifications include addition of cationic groups such as 4-amino-4-deoxy-L-arabinose (L-Ara4N) or phosphoethanolamine (pETN) that reduce the binding of colistin to the positively charged LPS due to electrostatic repulsion (Jeannot et al., 2017). Analysis of bacterial lipids using MALDI-TOF MS represents a valuable tool for detecting colistin resistance as it can directly identify the lipid modifications.

A MALDI-TOF MS method that enables rapid detection of colistin resistance in intact bacteria was recently developed (Dortet et al., 2018a). This method, named the MALDIxin test, employs the simple sample preparation procedure developed by Larrouy-Maumus et al. in which intact bacteria or the bacterial lysate arising from mild-acid hydrolysis (acetic acid 1%) are mixed with the special matrix on the MALDI target plate, followed by MALDI-TOF MS analysis in the negative ion mode (Larrouy-Maumus et al., 2016; Dortet et al., 2018a; Dortet et al., 2018b; Furniss et al., 2019; Dortet et al., 2020b). The acquired mass spectra enable differentiation of colistin-susceptible and colistin-resistant strains based on the mass-to-charge (*m*/*z*) of the major lipid A peaks. The addition of pETN to a phosphate group at position 1 of lipid A results in an *m*/*z* +123 shift of the native lipid A-related peak, while L-Ara4N modification causes an *m*/*z* +131 shift. This test can also discriminate between chromosome- and MCR-mediated resistance as L-Ara4N modification is generally observed in strains with chromosome-encoded resistance, while pETN modification predominates in strains expressing MCR (Dortet et al., 2018a; Sun et al., 2018; Moffatt et al., 2019). The efficiency of the MALDIxin test has been demonstrated for clinically relevant *E. coli*, *A. baumannii*, *K. pneumoniae* and *S. enterica* species, achieving detection of colistin resistance in less than 15 minutes (Dortet et al., 2018a; Dortet et al., 2018b; Dortet et al., 2019; Dortet et al., 2020a). Furthermore, this method was recently adapted for the routine MALDI Biotyper Sirius system increasing its clinical utility (Furniss et al., 2019).

Analysis of cellular fatty acid composition might also be employed to detect antibiotic-resistant strains in particular species. A study by Saichek et al. utilized MOLI MS analysis of fatty acids to discriminate between methicillin-resistant *S. aureus* (MRSA) and methicillin-susceptible *S. aureus* (MSSA) strains (Saichek et al., 2016). The differences in fatty acid profiles were observed, with greater prevalence of odd-numbered fatty acids in MSSA isolates and higher abundance of even-numbered fatty acids in MRSA isolates, which enabled differentiation of methicillin-susceptible and -resistant strains. Particularly important for therapeutic management, this method can simultaneously identify bacterial strains and detect antibiotic resistance (Saichek et al., 2016).

In addition to the applications to polymyxins and *S. aureus* cited above, more generally, lipids profiling by MS systems has the potential to be used for broad antibiotics susceptibility testing. For example, using MALDI coupled to high-resolution mass spectrometry named Fourier transform ion cyclotron resonance mass spectrometry (FT-ICR MS), Schenk and colleagues reported on the analogous distribution of altered fatty acids and glycerophospholipids, from *E. coli* strains, upon exposure to sublethal concentrations norfloxacin, an antibiotic that belongs to the class of fluoroquinolone antibiotics (Schenk et al., 2015). They concluded that variations of lipid content in the strain correspond to the extent of antibiotic resistance.

Indeed, that is supported by earlier work where significant alterations of fatty acid composition were observed in Gram-negative strains, such as *E. coli*, resistant to antibiotics such as tetracycline and polymyxins (Dunnick and O’Leary, 1970). The authors also reported on a correlation between the lipid content of Gram-positive *S. aureus* resistant to penicillin (Dunnick and O’Leary, 1970). Similar observations and correlations were also found for *Enterobacteriaceae*, such as *Serratia marcescens* (Chang et al., 1972; Suling and O’Leary, 1977).

However, further investigation is required to confirm the correlation between drug resistance, changes in membrane composition, and the relative abundance of different class of lipids, thus validating its use as an accurate and robust read-out of drug susceptibility routine MALDI-TOF MS system.

## Outlook and Areas for the Development of Lipid-Based Microbial Identification

**Figure 1:**
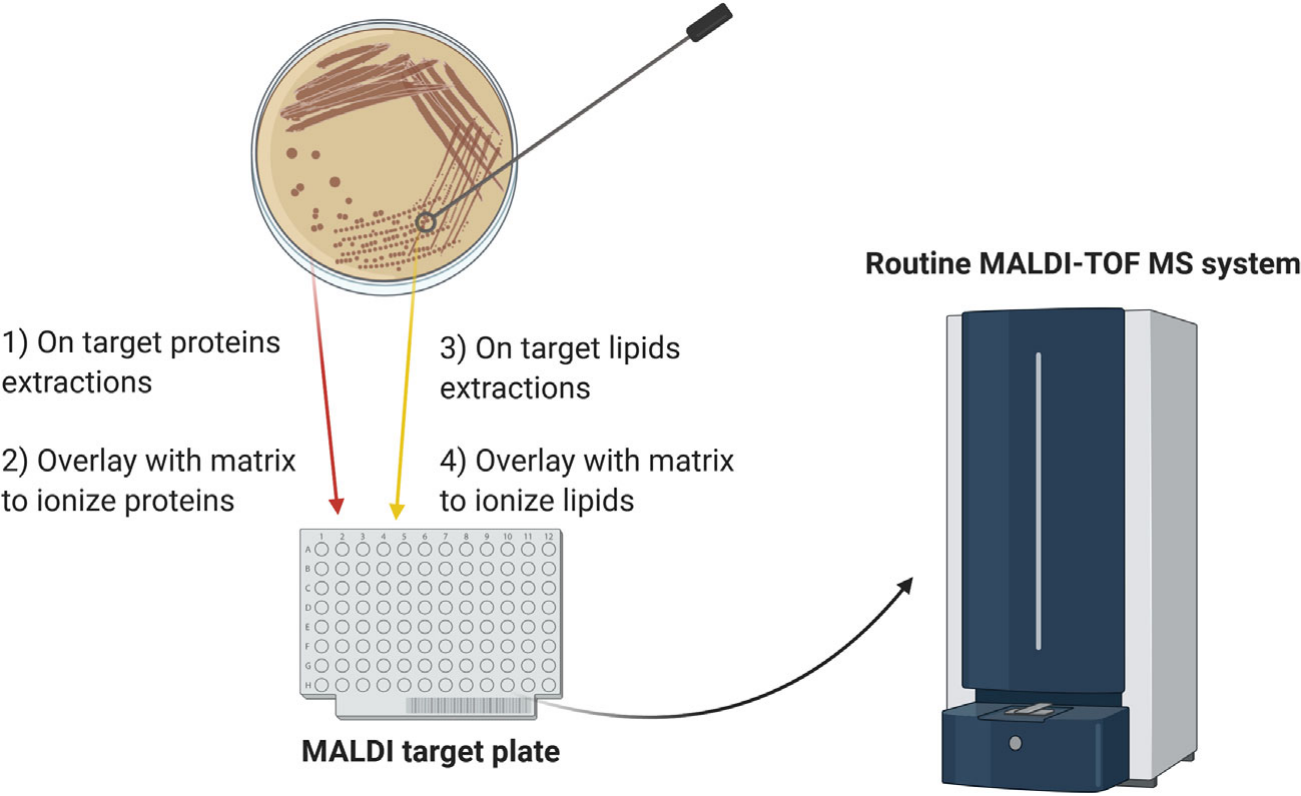
Schematic diagram of the proposed workflow for combining lipid and protein MALDI-TOF MS analysis. For protein analysis, a colony is smeared on the target plate and overlaid with matrix. For analysis of lipid A, heat-inactivated microorganisms are submitted to mild-acid hydrolysis and the membranes resulting from this treatment are deposited on an adjacent position on the target plate, followed by matrix addition. Alternatively, for mycobacteria and filamentous fungi, heat-inactivated colonies are directly smeared on the target plate and overlaid with the appropriate matrix. The combined approach can yield species- and subspecies-level microbial identification and detect antibiotic resistance in one analysis.

Adoption of lipidomics in clinical microbiology has lagged behind proteomic analysis due to the labor-intensive and time-consuming analytical methods initially available. However, novel methods of lipid analysis by MALDI-TOF MS have been developed in the last decade that can identify microorganisms with a speed and accuracy comparable to the routine MALDI-TOF MS analysis. Moreover, sample preparation has become significantly simpler and faster, particularly for lipid analysis of intact bacteria and filamentous fungi. Given these advances, lipid profiling has the potential to be employed alongside protein fingerprinting and may help to address some of the current constraints of MALDI-TOF MS, thereby further broadening its utility in clinical diagnostics and other routine microbiology applications. The workflow proposed in **Figure 1** would allow combination of lipid and protein analysis on the same MALDI target plate. This approach could accomplish several diagnostic goals in one analysis: the protein fingerprinting yields microbial identification at a species level while the lipid analysis can provide subspecies-level identification and detect antimicrobial susceptibility.

Although a considerable amount of progress has been made in optimizing sample preparation, further development of the bioinformatics resources is required to make the lipid analysis user-friendly in a clinical diagnostic setting, including the building of robust and accurate databases. Development of a representative lipid databases comparable in size and variety to the protein mass spectra libraries currently provided by commercial systems will improve the efficiency of microbial identification. Novel bioinformatic tools developed specifically for the analysis of lipid mass spectra such as the model-based spectral-library approach for matching mass spectra of glycolipids proposed by Ryu et al. will also advance identification (Ryu et al., 2019). A breakthrough in clinical microbiology diagnostics would be the ability to detect distinct microbial lipids directly from body fluids (e.g., serum, blood, and urine), thus avoiding the need for culture on agar plates or in liquid medium. Leung and colleagues attempted to address that challenge by spiking blood with *S. aureus* or *K. pneumoniae* B6 and recovering the bacteria by differential centrifugation to separate them from human cells. The lipids from the recovered bacteria were then extracted and analyzed by MALDI-TOF MS in the negative ion mode (Leung et al., 2017), and were able to get an excellent signal for 10^4^ CFU of bacteria spiked in blood followed by 6 hours incubation. Although promising, the experimental manipulations required to enrich in bacteria would represent a hurdle for the rapid turn-over of samples in clinical microbiology laboratories. Being able to enrich microbial species-specific lipids from human or animal fluids, for veterinary applications, with limited sample preparation will be a gamechanger in rapid microbial diagnostics.

In future, lipid analysis may be extended to detect resistance to other antibiotics as well as colistin. Several papers report that antibiotic-resistant strains, such as rifampin-resistant *M. tuberculosis* and daptomycin-resistant *E. faecalis*, display altered lipid composition (Mishra et al., 2012; Lahiri et al., 2016). By detecting these alterations and their relative abundances, lipid profiling may be able to identify the resistant microorganisms, however, this would require a large-scale analysis of the lipid composition which is a highly complex task if done manually. To facilitate the data analysis, machine learning techniques should be developed to discriminate the mass spectra of antibiotic-resistant and -susceptible strains as has already been done in several studies employing protein-based MALDI-TOF MS analysis (Weis et al., 2020).

## Author Contributions

VS, GL-M, and MK conceived the review and wrote the manuscript. All authors contributed to the article and approved the submitted version.

## Funding

This study was supported by the MRC Confidence in Concept Fund and the ISSF Wellcome Trust grant 105603/Z/14/Z (GL-M).

## Conflict of Interest

GL-M is a co-inventor of the MALDIxin test for which a patent has been filed by Imperial Innovations. MK is an employee of Bruker, the manufacturer of the MALDI Biotyper system.

The remaining author declares that the research was conducted in the absence of any commercial or financial relationships that could be construed as a potential conflict of interest.
